# Exploring the relationship between physical activity, life goals and health-related quality of life among high school students: a cross-sectional study

**DOI:** 10.1186/s12889-016-3407-0

**Published:** 2016-08-03

**Authors:** Julie Sigvartsen, Leiv Einar Gabrielsen, Eirik Abildsnes, Tonje H. Stea, Christina Sandvand Omfjord, Gudrun Rohde

**Affiliations:** 1 Faculty of Health and Sport Sciences, University of Agder, Kristiansand, Norway; 2 Department for Child and Adolescent Mental Health, Sorlandet Hospital Health Enterprise, Kristiansand, Norway; 3 Department of Global Public Health and Primary Care, University of Bergen, Bergen, Norway; 4 Department of Public Health, Sport and Nutrition, Faculty of Health and Sport Sciences, University of Agder, Kristiansand, Norway

**Keywords:** Adolescents, High school, Health-related Quality of Life, Life Goals, Physical Education, Physical Activity

## Abstract

**Background:**

Two models were developed to increase high school students’ participation in physical education (PE): “motion enjoyment” and “sport enjoyment”. The first model focuses on increasing knowledge about the health benefits of a physically active lifestyle and thereby promoting a positive attitude towards physical activity, whereas the second model focuses on techniques and practices for enhancing athletic performance. The aims of the present study are to investigate and understand the similarities and differences between students selecting “motion enjoyment” vs. “sport enjoyment” and to examine the extent to which life goals and reported physical activity are associated with health-related quality of life (HRQOL).

**Method:**

A total of 156 high school students (mean age, 16 years [standard deviation = 0.8], 123 girls and 33 boys) were included in this cross-sectional study. HRQOL and life goals were measured using KIDSCREEN-10 and the Adolescent Life Goal Profile Scale, respectively. Physical activity was measured using a self-reporting questionnaire intended to describe the students’ leisure-time activity. Independent sample *t*-tests, chi-square, one-way analyses of variance and multiple regression analysis were applied.

**Results:**

Self-reported physical activity level and HRQOL were higher among students in the “sport enjoyment” program, while the perceived importance of life goals was the same regardless of the preferred PE model. Multiple regression analyses revealed that the perceived importance of relations-oriented life goals (B = −5.61; 95 % confidence interval CI = −10.53 to −0.70; *p* = .026), perceived importance of generativity-oriented life goals (B = 4.14.; 95 % CI = 0.85 to 7.422; *p* = .014), perceived attainability of relations-oriented life goals (B = 7.28; 95 % CI = 2.49 to 12.07; *p* = .003), age (B = −7.29; 95 % CI = −11.38 to −3.20; *p* = .001) and gender, with boys as the reference group (B = −12.10; 95 % CI = −19.09 to −5.11; *p* = .001), were independently associated with increased HRQOL. In exploring the relationships of self-reported physical activity during leisure time, stage of change (B = 3.53; 95 % CI = 1.49 to 5.51; *p* = .001), gender, with boys as the reference group (B = −8.90; 95 % CI = −15.80 to −2.00; *p* = .012), and age (B = −6.62; 95 % CI = −10.57 to −2.66; *p* = .001) were independently associated with increased HRQOL.

**Conclusion:**

Self-reported physical activity habits and life goals were associated with HRQOL to a limited extent. However, the perceived importance of life goals appears to reflect other aspects of individual well-being than HRQOL.

## Background

Regular physical activity is beneficial to the physical, mental and social aspects of health among adolescents [1–3]. Physical activity in this study is defined as “any bodily movement produced by skeletal muscles that results in energy expenditure” [4]. Data from previous studies show that a high level of physical activity in childhood and adolescence predicts a high level of physical activity in adulthood [5, 6]. However, several studies have documented decreasing physical activity among adolescents [7, 8], a trend also evident in Norway [9, 10]. Studies have also reported that health-related quality of life (HRQOL) declines with age during adolescence, with the decline being larger among girls than boys [11–13]. Furthermore, engaging in regular physical activity in adolescence is associated with a higher HRQOL [14].

Physical education (PE) programs in high school have the potential and intention to inspire adolescents to achieve a lifelong enjoyment of physically active behavior in all aspects of life [15]. The self-determination theory suggests that human motivation is based on fundamental psychological needs: autonomy, perceived competence and relatedness [16]. Considering this, a PE program based on these principles has the potential to improve adolescents’ subsequent physical activity habits and increase students’ internal motivation to remain active [17–19]. The empirical approach to the study of human strengths and virtues often includes assessing choices of life goals, as this knowledge can be used to promote health and well-being, which are key issues for optimal human functioning [20]. Locke [21] noted that “goals are the means by which values and dreams are translated into reality”. Becoming aware of one’s intrinsic life goals appears to be vital in the pursuit of meaning, and adolescents who have clearly defined goals and believe they are attainable exhibit increased mental health, well-being, and self-efficacy [22].

In Norway, PE is compulsory, and students have to pass physical education standards to receive their high school diploma. From a health perspective, it is also desirable for the students to make use of the experiences they gain in PE to experience a lifelong enjoyment of physical activity. With the intention to increase participation in PE and to involve students who appreciate sports and competition as well as those who do not, two models were developed in this study: a “motion enjoyment” and a “sport enjoyment” model. “Motion enjoyment” emphasizes the promotion of positive PE experiences and teaching students about the positive health effects of being physically active. The other model, “sport enjoyment”, presents athletic sports, activity skills, techniques and improvement of physical fitness. Competitions and tests were emphasized in this model.

The aims of the present study are to investigate and understand the similarities and differences between students selecting the “motion” or the “sport enjoyment” model and to examine the extent to which life goals and reported physical activity are associated with HRQOL. This knowledge will provide insight into the basic psychological characteristics, differences and similarities of these student cohorts and how these factors relate to their preferred PE options. We believe that this understanding may be instructive and useful when defining future PE programs, both on a practical and a motivational level.

## Methods

### Study design, setting and participants

To examine the characteristics of the students who select different directions in PE, cross-sectional baseline data were used. The target group was students who were in their 1st year of vocational studies at two high schools in Southern Norway. This target group was selected based on the teachers’ experiences of low student commitment to participating in PE, especially among these students. Low commitment was defined as low attendance and low engagement and enthusiasm to PE. The low commitment to PE in this group was also evident in the school’s statistics [23]. To increase participation in PE, the two high schools involved in this study adopted the two PE models, “motion enjoyment” and “sports enjoyment”. In other words, all students were asked to choose one of the two PE models. The study lasted one school year (August 2013 to June 2014). In total, 181 (82 %) first-year students of 220 possible students answered the questionnaires in the study. A total of 25 students aged 19 years or older were excluded, as the instruments measuring life goals and HRQOL have not been validated for this age group. The post-data were not included in this study because half of the sport enjoyment students were missing at the time of data collection.

In Norway, most students attend public schools, and after finishing junior high school (at the age of 15–16 years), all adolescents are entitled to attend high school. High schools in Norway are organized into twelve different programs: three programs for general studies and nine programs for vocational educational programs [24]. Students in vocational programs were the target group of the models implemented, and they represent the study population of the present study. All students in the selected educational programs were invited to participate in the study.

During their first week of high school, students received information about the PE models at meetings, via the student intranet and by written information. After six weeks, which represented an adaption period to the new school and school system, the students were required to decide which PE model group they wanted to join. The total sample of 156 students comprised 123 girls (78 %) and 33 boys (22 %). The mean age was 16 years (standard deviation [SD] = 0.8; range, 15–18 years). The students attended the following high school vocational programs: Restaurant and Food Processing (26 %), Design, Arts and Craft (27 %) and Health Care, Childhood and Youth Development (47 %). The majority selected “motion enjoyment” (69 %), while the remaining participants selected “sport enjoyment” (31 %).

### Instruments and variables

The students responded to an online questionnaire (SurveyXact®) containing several well-validated items. The questionnaire took 20–30 min to complete. Participation required written consent.

#### Demographic variables

The demographic data included gender, age, choice of PE model, choice of vocational program and parents’ socio-economic status. For the analysis, the students were divided into two age groups: 15–16 years and 17–18 years. Parents’ socio-economic status was assessed by education level and was divided into three groups: education level less than 13 years, education level more than 13 years, and education level unknown to the students.

#### Self-reported physical activity behavior

The students were asked to describe how many times a week and the number of hours in their leisure time that they were physically active to the point that they were out of breath or were sweating. In Norway, all organized PA (e.g., soccer, gymnastics) takes place outside the school system. Furthermore, the students reported their level of enjoyment of PE and previous grade in PE. Screen time was reported as an indication of sedentary behavior. All the self-reporting physical activity questions have previously been used and validated in the study “Youth with Profits” [25]. To describe the students’ starting point for further exercise and to provide an account of students who may be in different stages regarding exercise behavior, the Stages of Change questionnaire was used. This questionnaire uses a clear exercise definition [26] and describes each of the five stages (pre-contemplation, contemplation, determination, action and maintenance) with a sentence. The Stages of Change questionnaire is regarded as a valid method to describe and modify exercise behavior [27]. In accordance with previous studies, we chose to express the Stages of Change data as continuous variables [28].

#### General health perception

The students evaluated their general health by answering the question “on a regular day, how would you describe your health?” on a five-point Likert scale ranging from excellent to poor.

#### Life goals

Life goals were measured using the Adolescent Life Goal Profile Scale (ALGPS). The ALGPS provides information about the perceived importance and perceived attainability of the four most subscribed-to life goals for finding meaning. The life goals are considered the “Big 4” of meaning [29] and are, when adapted to adolescents: Relations, Generativity, Religion and Achievements. The scale can be applied to general adolescent research and as an approach to individual therapy in mental health services [22]. The scale consists of 32 items (16 on perceived importance of life goals and 16 on perceived attainability of life goals) scored on a five-point Likert scale ranging from “not important” to “very important” and from “not attainable” to “very attainable,” respectively. Scoring the ALGPS leaves us with eight independent variables (range, 0–5), perceived importance of each of the four life goals and perceived attainability of these life goals [22]. The ALGPS questionnaire was developed and validated in Norway [22]. Cronbach’s alphas of the four domains in this study were 0.74 for relations, 0.80 for generativity, 0.47 for religion and 0.80 for achievements. The religion alpha value was quite low, and we decided not to include this life goal in our analysis.

#### Health-related quality of life

The Norwegian version of the KIDSCREEN-10 was used to measure HRQOL [30]. This questionnaire is a generic, multidimensional construct with 10 questions covering perceptions of physical, emotional, mental, social and behavioral components of well-being during the last week on a five-point response scale [31]. According to the scoring procedures, KIDSCREEN-10 provides a general HRQOL index of the ten components expressed as a value from 0 to 100, with 100 representing excellent HRQOL [31]. This questionnaire has shown satisfactory reliability and validity and has been thoroughly tested for assessing the psychometric properties of the 8–18-year-old age group in several countries, including Norway [30–33]. In this study, Cronbach’s alpha of the HRQOL index was 0.74, which is considered satisfactory [34].

### Statistical analysis

The statistical analysis was carried out using the Statistical Package for Social Sciences (IBM SPSS Statistics 21®; IBM, Armonk, NY, USA). The descriptive statistics are presented as numbers, percentages (%), means and SDs. In comparison groups, independent sample *t*-tests were used for continuous variables, crosstabs for categorical variables, and Mann–Whitney *U* tests for non-normally distributed continuous variables.

Multiple regression analysis with a multiple backward elimination method was applied to further analyze the association between life goals and HRQOL and between self-reported physical activity variables and HRQOL. The independent variables in the multiple analyses were chosen based on the univariate analyses, experiences with groups of adolescents and associates of HRQOL in previous studies. A *p*-value less than .05 was considered statistically significant [35].

## Results

The demographic characteristics of the students participating in the two PE models are presented in Table 1. The majority of the boys (63 %) participated in “sport enjoyment” (*p* < .001), whereas a majority of the girls (78 %) participated in “motion enjoyment” (*p* < .001).

**Table 1:** Mean scores for students selecting “motion” or “sport enjoyment” and comparison between the groups regarding demographics, general health perceptions and physical activity habits

Number (%) or mean (± SD)	Total (*n* = 156)	“Motion enjoyment” (*n* = 108)	“Sport enjoyment” (*n* = 48)	*P*-value
Age (years)^a^	16.1 (0.6)	16.2 (0.7)	16.0 (0.5)	.229
Boys	33 (21 %)	12 (11 %)	21 (44 %)	**< .001**
Girls	123 (79 %)	96 (89 %)	27 (56 %)	
Vocational programs^b^				
Health Care, Childhood and Youth Development	73 (47 %)	58 (54 %)	15 (33 %)	**.004**
Design, Arts and Crafts	41 (27 %)	30 (28 %)	11 (24 %)	
Restaurant and Food Processing	40 (26 %)	20 (18 %)	20 (43 %)	
Education level^c^				
Father above 13 years	27 (19 %)	16 (15 %)	10 (24 %)	**.421**
Father under 13 years	57 (39 %)	41 (40 %)	16 (39 %)	
Father “don’t know”	61 (42 %)	46 (45.0 %)	15 (37 %)	
Mother above 13 years	31 (20 %)	16 (16 %)	14 (37 %)	**.042**
Mother under 13 years	59 (41 %)	44 (48 %)	14 (34 %)	
Mother “don’t know”	55 (39 %)	43 (42 %)	12 (29 %)	
General health perception	3.8 (0.9)	3.6 (0.9)	4.1 (0.9)	**.011**
Physical activity habits				
Physical activity level in leisure (number/day)	2.3 (2.0)	2.0 (2.0)	2.9 (1.8)	**.013**
Physical activity level in leisure (hours/day)	2.7 (1.5)	2.4 (1.4)	3.5 (1.7)	**.001**
Like PE	4.3 (2.0)	3.8 (1.9)	5.4 (1.7)	**< .001**
Screen time (hours)	4.9 (1.5)	5.1 (1.3)	4.2 (1.7)	**.005**
Grade; PE	3.3 (1.6)	3.1 (1.5)	3.8 (1.7)	**.035**
Stage of change	3.0 (1.3)	2.8 (1.2)	3.7 (1.4)	**< .001**

Continuous variables are expressed as mean and standard deviation (SD). Categorical variables are expressed as number and percentage (%). In the group comparison, independent sample *t*-tests were used for continuous variables and chi-squared tests were used for categorical variables. Bold numbers indicate *p*<0.05

^a^Age is non-normal in distribution, and is expressed as mean and standard deviation (SD); in the group comparisons, Mann–Whitney *U* tests were used

^b^Vocational programs: Three students did not report which program they attended (missing data)

^c^Educational level father/mother presented as number and percentage (%) with education level above/under 13 years; don’t know signifies the students who were unsure of parents’ education levels

The majority of the students in Health Care, Childhood and Youth Development (80 %, *n* = 58) and Design, Art and Craft (73 %; *n* = 30) selected “motion enjoyment” (*p* = .002). On the other hand, approximately half of the students attending Restaurant and Food Processing (*n* = 20) selected “sport enjoyment”. Most of the students attending either Health Care, Childhood and Youth Development or Design, Art and Craft were girls (88 % and 98 %, respectively). In contrast, 43 % of the students attending Restaurant and Food Processing were girls.

### Adolescents’ physical activity habits

The students participating in “sport enjoyment” reported that they were physically active three times a week during their leisure time, whereas the students participating in “motion enjoyment” reported that they were physically active twice a week (*p* = .013). The students participating in “sport enjoyment” reported that they were physically active 5–6 h a week on average, whereas the students participating in “motion enjoyment” were physically active 3–4 h a week on average (*p* = .001). Additionally, the students participating in “sport enjoyment” reported spending less hours on screen time behavior (*p* = .005), and they reported liking PE more *(p* = .001) than those participating in “motion enjoyment”. There were significant differences in the students’ readiness to change PA habits, which is expressed by stage of change, with students participating in “motion enjoyment” reporting lower scores than the students participating in “sport enjoyment” (*p* < .001) (Table 1).

### Health-related quality of life (HRQOL)

The mean HRQOL score of all the students was 62.0 [SD = 17.1]. There was a significant difference in HRQOL (Table 2) between students participating in “sport enjoyment” and students participating in “motion enjoyment” (mean = 68.3 [SD = 15.2] vs. mean = 59.4 [SD = 17.1], respectively, *p* = .003).

**Table 2:** Mean scores and standard deviations in health-related quality of life (HRQOL) and Adolescent Life Goals Profile Scale (ALGPS) in “motion” and “sport enjoyment”

Number (%) or mean (± SD)	Total (*n* = 156)	“Motion enjoyment”(*n* = 108)	“Sport enjoyment” (*n* = 48)	*P*-value
HRQOL	62.1 (17.1)	59.6 (17.1)	68.6 (15.2)	**.003**
ALGPS				
Perceived importance				
Relations	3.7 (0.9)	3.8 (0.9)	3.6 (0.8)	.417
Generativity	3.6 (0.9)	3.6 (0.9)	3.5 (1.0)	.485
Achievement	3.3 (1.0)	3.3 (1.0)	3.3 (1.0)	.774
Perceived attainable				
Relations	3.7 (0.8)	3.7 (0.8)	3.7 (0.8)	.887
Generativity	3.3 (0.8)	3.3 (0.8)	3.3 (0.7)	.619
Achievement	3.1 (0.8)	3.1 (0.8)	3.1 (0.9)	.655

Continuous variables are expressed as mean and standard deviation (SD). In the group comparison, independent sample *t*-tests were used. HRQOL was measured with KIDSCREEN-10 (range 0–100). ALGPS measures life goals. Bold numbers indicate *p*<0.05

### Adolescent life goal profile scale (ALGPS)

The results showed no significant differences in perceived importance and perceived attainability of life goals between those participating in “motion” and those in “sport enjoyment” (Table 2). Relations (mean = 3.7 [SD = 0.9]) was rated as the most important life goal.

### HRQOL associates

The multiple regression analysis (backward elimination) explored the association between life goals and HRQOL, adjusted for age, gender and PE model. Perceived importance of relations, perceived importance of generativity, perceived attainability of relations, decreased age and gender (with boys as the reference group) were significantly associated with increased HRQOL (Table 3).

**Table 3:** Association between life goals, demographics and HRQOL in high school students

	Full model			Final model		
Demographic	Adjusted B	95 % CI	*P*-value	Adjusted B	95 % CI	*P*-value
Girls	−9.85	−17.47 to −2.23	**.012**	−12.10	−19.09 to −5.11	**.001**
Boys	Ref			Ref		
Age	−6.89	−11.08 to −2.71	**.001**	−7.29	−11.38 to −3.20	**.001**
“Motion enjoyment”	−4.57	−10.76 to 1.61	.146			
“Sport enjoyment”	Ref					
ALGPS						
Perceived importance						
Relations	−3.77	−9.36 to 1.82	.184	−5.61	−10.53 to −0.70	**.026**
Generativity	3.01	−2.61 to 8.63	.291	4.14	0.85 to 7.42	**.014**
Achievements	−0.68	−5.13 to 3.76	.761			
Perceived attainable						
Relations	4.54	−1.33 to 10.41	.129	7.28	2.49 to 12.07	**.003**
Generativity	0.36	−6.19 to 6.90	.915			
Achievements	3.38	−1.88 to 8.65	.206			
Adjusted *R* ^2^	20.1 %			19.6 %		

Adjusted unstandardized regression coefficient, 95 % CI, *p* values and *R* ^2^ for the full and final model using multiple backward regression analysis. Dependent variable: HRQOL. All variables are continuous variables except gender and PE model. Bold numbers indicate *p*<0.05

Multiple regression analysis with a multiple backward elimination method was also applied to further analyze which self-reported physical activity variables were independently associated with HRQOL, adjusted for age, gender, vocational program and PE model (Table 4). The backward method revealed that stage of change, decreased age and gender (with boys as the reference group) were significantly associated with increased HRQOL.

**Table 4:** Association between self-reported physical activity, demographics and HRQOL in high school students

	Full model			Final model		
Demographic	Adjusted B	95 % CI	*P*-value	Adjusted B	95 % CI	*P*-value
Girls	−5.10	−13.00 to 2.79	.203	−8.90	−15.80 to – 2.00	**.012**
Boys	Ref			Ref		
Age	−5.73	−9.77 to −1.70	**.006**	−6.62	−10.57 to −2.66	**.001**
Health Care, Childhood and Youth Development/Design, Arts and Crafts	−2.27	−5.64 to 1.10	.184			
Restaurant and Food Processing	Ref					
“Motion enjoyment”	0.77	−8.12 to 4.00	.637			
“Sport enjoyment”	Ref					
Like PE	0.75	−0.80 to 2.34	.335			
Physical activity in leisure; (number)	0.95	−0.85 to 2.74	.299			
Stage of change	1.67	−1.26 to 4.60	.261	3.53	1.49 to 5.51	**.001**
Screen time	−1.01	−2.85 to 0.83	.279			
Adjusted *R* ^2^	19.1 %			18.1 %		

Adjusted unstandardized regression coefficient, 95 % CI, *p* values and *R* ^2^ for the full and final model using multiple backward regression analysis. Dependent variable: HRQOL. All variables are considered as continuous except gender, vocational programs and PE model. Bold numbers indicate *p*<0.05

## Discussion

Adolescents who selected “sport enjoyment” reported themselves as more physically active than those selecting “motion enjoyment”, and they also reported a higher HRQOL. There were no significant differences in life goal factors between the students in the “motion” or “sport enjoyment” programs.

The aim of the present study is to investigate and understand the similarities and differences between students selecting “motion” and those selecting “sport enjoyment”. In this study, the students had the opportunity to participate in their preferred PE model, and a minority of the students selected “sport enjoyment”. The results show that self-reported physical activity level and HRQOL were higher among the “sport enjoyment” students. Although physical activity is generally understood as being beneficial to health, many individuals find it challenging to maintain a high level of physical activity, most notably because of a lack of motivation and a lack of the required skills and knowledge [3]. Being able to choose between different PE approaches should facilitate one’s autonomy and the development of internal motivation to engage in a physically active lifestyle and experience the psychosocial benefits of physical activity [36]. Activities that are internally rewarding are more likely to be repeated [37]. As a result, when a choice is offered that meets the student’s needs regarding autonomy, competence and relatedness to a greater extent, it will most probably increase their self-determination, learning and well-being [38]. Previous research has identified that self-determined behavior [16] might contribute to increasing the quality of motivation, probably also in PE. Additionally, an emphasis on facilitating self-determination in physical activity is likely to increase an individual’s intention to be active during leisure time, both pre- and post-graduation [17–19], and to comply with the intentions of PE programs. It appears that teachers’ use of intrinsic goals to frame learning activities and their provision of autonomy-supportive learning climates have significant effects on students becoming more dedicated, more genuinely engaged in learning activities and experiencing more enjoyment [39]. Thus, the use of intrinsic goals has important implications for designing optimal learning environments [40].

According to Granero-Gallegos et al. [41] and Camacho-Miñano et al. [42], it is important that the selected PE activity should generate fun, enjoyment, satisfaction and interests. High school-based interventions have previously demonstrated a positive impact on the subsequent duration of physical activity levels [43–46]. Furthermore, boys seem to benefit more than girls from such interventions, and interventions targeting girls’ preferences should therefore be offered [42, 46]. In this sample, physical activity decreased with age, and boys were more physically active than girls. The girls tended to prefer “motion enjoyment”, thus focusing on elements from this perspective could help inspire girls to be more active. In short, understanding why some adolescents are physically active while others are not is crucial to precisely targeting those factors that are known to cause inactivity [47, 48].

In the present study, students who reported that they enjoyed PE also reported higher HRQOL. Adolescence is a developmental period that involves a range of psychosocial and physiological changes, and trying to address developing bodies and identities may directly lead to situations that affect HRQOL [49]. Several other studies have documented gender differences in HRQOL among adolescents [12, 13], and gender was found to be a predictor of HRQOL in the adjusted analysis in this study using boys as the reference group. Furthermore, age was significantly associated with HRQOL in the multiple regression analysis, with older adolescents scoring lower on HRQOL. These findings are in line with earlier studies in the field [11–13].

Although a majority of the students preferred “motion enjoyment” to “sport enjoyment” in this study, the perceived importance of life goals was equally valued among students in both groups. In a recent study that compared a clinical population with a non-clinical sample, Gabrielsen, Watten and Ulleberg [50] found that adolescents with mental health problems retained most of their life goals and found these to be equally important as others. There were no differences observed in the perceived importance of generativity-, religion- and achievement-oriented life goals. However, the clinical sample reported a lower quality of life and self-efficacy and also a lower perceived attainability of their goals [50]. Individuals who frequently exercise spend significantly more time exercising and value exercise goals more highly than less-frequent exercisers. It is very important to note that even when these goals appear to be valued differently, the time spent on other goals not including exercise is valued the same [51].

The students who selected “motion enjoyment” reported lower HRQOL scores than those in the “sports enjoyment” group, but they maintained clear goals of what their life should be like. The multiple regression analysis showed that the perceived importance of relation-oriented life goals, the perceived importance of generativity-oriented life goals and the perceived attainability of relations-oriented life goals adjusted for demographic variables were associated with increased HRQOL. In other words, it is fair to assume that what really separates the two groups is their perceived self-efficacy, particularly in regard to *performing* in sports. Clearly, if individuals do not consider their sports performance to be acceptable, it is unlikely they will enter a program that emphasizes exactly this. Determining the cause of reduced self-efficacy is a tall order, as the underlying factors may range from early psychosocial life experiences to a culturally conditioned reduced body image. Still, knowing one’s strengths and what situations to avoid (here, this might mean sports enjoyment) is crucial for maintaining strong psychological health in the long term [52, 53].

### Strengths, limitations and future perspectives

All the results are based on self-reported data, which inherently imply issues of validity. However, the questionnaires used were all validated for the age cohort in this study [25]. Life goal profiles were measured using the ALGPS. Measuring life goals provides valuable information about the respondents’ inner values and thoughts. Regarding the strong connections between the choice of goals and psychological functioning, the results of the ALGPS help provide insight into the HRQOL findings. The KIDSCREEN-10 presents only one summary score when measuring HRQOL; this may be an oversimplification of something as complex as quality of life. A more informative result might be found if the 27- or 52-item versions were used. However, the 10-item version has been validated in screening children and adolescents (aged 8–18 years) [30, 32]. Additionally, a conceptual definition of HRQOL was clearly described. Research has shown that children understand and reflect on what happens in their life from at least the age of eight, and the reliability of self-reports on their health and well-being is high [52].

This cross-sectional study has limitations with regard to the interpretation of the data. Specifically, it is not possible to infer causal changes over time. However, it would be challenging to predict changes in the phenomena of HRQOL and life goals and to link such changes to participation in either the motion or sport enjoyment groups in a longitudinal study. The causal discussions presented here are founded on the theoretical background of the study and previous research. These high schools were selected based on the teachers’ experiences of low student commitment to PE as well as the corresponding statistics from the schools. Accordingly, there were some limitations regarding sample size and gender distribution. The gender differences may be related to the topics of the vocational programs presented, with the majority of girls attending Health Care, Childhood and Youth Development and Design, Art and Craft. Additionally, some limitations regarding the distribution between gender and student preferences of “motion enjoyment” or “sport enjoyment” are present. Inequalities were observed between the models of PE, in which 70 % of the students enrolled in motion enjoyment, whereas 30 % enrolled in sport enjoyment. Furthermore, 25 students were excluded from the analysis due to their age being older than 19 years. The distribution and sample size of the students present some challenges regarding the generalization of the findings.

Longitudinal studies on HRQOL and ALGPS are warranted, and it would be interesting to document, particularly within this age cohort, possible changes in quality of life, perceived importance of life goals, and a range of other variables including physical activity and self-perception.

## Conclusions

Students who chose to participate in “sport enjoyment” reported engaging in more leisure time physical activity and experienced a higher HRQOL than their peers who preferred “motion enjoyment”. Nonetheless, life goals were perceived as equally important by the two groups, indicating substantial similarities in their basic psychological functioning. However, life goals and self-reported physical activity showed few and limited associations with HRQOL in the adjusted model. It is essential to note that even if HRQOL and the pursuit of life goals increase subjective well-being, they are not rated similarly among the students. Thus, even though HRQOL and ALGPS explore some similar life domains, the value placed on life goals reflects other aspects of individual functioning besides the subjective experience of HRQOL.

## Abbreviations

ALGPS, adolescent life goals profile scale; HRQOL, health-related quality of life; PE, physical education
